# Editorial: Efforts to reduce feed-food competition

**DOI:** 10.3389/fvets.2023.1335007

**Published:** 2023-12-05

**Authors:** Claudio Forte, Domenico Pietro Lo Fiego, Massimo Trabalza Marinucci, Davide Pravettoni, Antonio Natalello

**Affiliations:** ^1^Department of Veterinary Sciences, University of Turin, Turin, Italy; ^2^Department of Life Sciences, University of Modena and Reggio Emilia, Reggio Emilia, Italy; ^3^Department of Veterinary Medicine, University of Perugia, Perugia, Italy; ^4^Department of Veterinary Medicine and Animal Sciences, University of Milan, Lodi, Italy; ^5^Department of Agriculture, Food and Environment (Di3A), University of Catania, Catania, Italy

**Keywords:** sustainability, circular economy, livestock, former foodstuff, insects, fermented feed

Livestock is facing numerous challenges concerning safety and security, climate change and environmental emissions, and public opinion about animal welfare and sustainability. Moreover, fluctuations in the international market economy due to the recent conflicts in Ukraine and the Middle East drive the whole chain of production toward a necessary increase in sustainability and circularity.

Feed-food competition has been defined as “*the tensions and trade-offs between using edible crops and other resources to either feed people directly or feed livestock*” (1). Increasing the recovery of critical raw materials is one of the key challenges in the transition to a more circular and resilient economy. In this scenario, feed-food competition is gaining attention, and researchers agree that alternatives to unsustainable feed ingredients need to be explored.

The main objective of the Research Topic was to collect data focused on reducing feed-food competition in a trans-disciplinary approach. In this Research Topic, five scientific articles have been published.

Insects are now recognized worldwide as a sustainable feed ingredient because they can utilize organic waste as a substrate for breeding, and the meal produced from them has been demonstrated to be an effective replacement for both soybean and fish meal. In their review, Belhadj Slimen et al. provided an overview of the use of the most studied insect species in poultry feed, focusing on nutritional value and digestibility, effects on live performance, product quality and health. Concerns about their safe use as feed ingredients have been addressed as well. Even if insects are acknowledged as a valuable source of proteins, MUFA, PUFA, minerals, and vitamins in feeds for poultry, authors recognize the need for a reduction of production costs and for strict control of contamination and spreading of pathogens. Nonetheless, more research on the role of antimicrobial peptides in association with chitin and chitosan should be performed to deepen the knowledge about their antimicrobial potentialities.

The study proposed by Bongiorno et al. explores the possibility of using live black soldier fly larvae as environmental enrichment in medium-growing chickens and their effects on *in vivo*, blood parameters, and slaughter performance. In this experiment, 240 male and female Label Naked Neck birds (21 days of age) were allotted to four experimental groups, according to sex and diet, and reared following European rules for organic farming (2). The results showed benefits deriving from the live larvae administration on *in vivo* performance more evident in early phases and in males compared to females. An interesting result has been achieved in gamma-glutamyl transferase concentration in the blood resulting in lower (*P* < 0.05) in supplemented groups compared to controls; authors indicate the chitin content of the larvae as possibly responsible for this positive outcome. Bursa of Fabricious and spleen resulted bigger in treated animals, suggesting a positive effect on immune functions.

Natural feed additives able to improve performance and feed utilization can represent an alternative to reduce feed food competition. In the study presented by Liu et al., Ganoderma lingzhi culture, a medicinal fungus, has been used as fermented feed for Sanhuang broilers; the aim was to explore its impact on growth performance, serum biochemical profile, meat quality, intestinal morphology, and gut health. A total of 192 chickens were allocated in the four experimental groups (112 days old, six replicates per group with eight broilers/pen). Authors recorded better performance in terms of FCR at the end of the trial, an increase in SOD and HDL serum levels and improved SCFA caecal concentrations. Accordingly, a higher diversity resulted from microbiota analyses, with a higher number of SCFAs-generating bacteria in the gut. It appears that the administration of fermented feed could positively affect the slow-growing chicken livestock system posing a new basis for developing biological feeds based on medicinal fungi fermentation.

As already stated, in recent years, many factors affected the availability of commonly used raw materials in animal nutrition. One of these cases is represented by fishmeal. Overfishing, climatic changes, and economic issues caused a worldwide systemic decline in fish meal availability. This pushed aquaculture to search for new and more sustainable feed ingredients. Lin et al. published a study aimed at substituting fish meal with corn gluten meal, a by-product of maize processing, for pacific white shrimp nutrition. In their experimental design corn gluten meal was included in six dietary groups up to 60% (0%, 10%, 20%, 30%, 40%, and 60%) replacing fish meals, which were decreased from 300 g^*^kg^−1^ in the 0% group to 120 g^*^kg^−1^ in the 60% group. The authors analyzed growth, feed utilization, enzymatic activity, and nutrient digestibility in juvenile subjects. The higher replacement rate negatively affected the shrimp performance; the authors attribute these effects to a disbalance in amino acids of the feed. Interestingly, based on broken line regression analysis an optimal dietary corn gluten meal replacement was found at 27.47% of the total protein derived from fish meal.

Of the proposed solutions to face feed food competition, the use of former foods (FF) in animal feed constitutes a new area of research (1). FFs are “*foodstuffs*, […] *manufactured for human consumption in full compliance with the EU food law, but which are no longer intended for human consumption* […] *and which do not present any health risks when used as feed*” (3), Research proposed by Vastolo et al. investigated the nutritional characteristics of four different types of pasta to be included in pig diets. The authors analyzed the chemical composition and digestibility of pasta comparing the value obtained with barley as reference material. All the analyzed types of pasta (wholemeal, semolina, purple, and tricolor) resulted in higher protein and energy content (*P* < 0.001) and lower protein (*P* < 0.001) compared to barley. All the tested products showed higher values of resistant starch compared to barley, which was mainly composed of slowly digestible starch. The authors stated that the examined FFs seem suitable for swine nutrition, but the different starch digestibility should be considered.

In summary, the presented research articles present new insights for the further development of a more sustainable livestock system, able to reduce the feed food competition and face the growing interest of new consumers.

## Author contributions

CF: Conceptualization, Writing – original draft, Writing – review & editing. DL: Writing – original draft. MT: Writing – original draft, Writing – review & editing. DP: Writing – original draft. AN: Writing – original draft, Writing – review & editing.

## References

[1] Breewood H Garnett T. What is Feed-food Competition? (Foodsource: building blocks). Oxford: Food Climate Research Network, University of Oxford (2020).

[2] European Commission. Commission Regulation (EC) N° 889/2008 of 5 September 2008 laying down detailed rules for the implementation of Council Regulation (EC) No 834/2007 on organic production and labelling of organic products with regard to organic production, labelling and control. Off J Eur Union. (2008) 250:1–84.

[3] Regulation (EU) No 2017/1017 of 15 June 2017 Amending Regulation (EU) No 68/2013 on the Catalogue of Feed Materials. Available online at: https://eur-lex.europa.eu/legal-content/EN/TXT/?uri=CELEX%3A32017R1017 (accessed November 3, 2023).

